# A Comparative Study of CCM and CCS Membrane Electrode Assemblies for High-Temperature Proton Exchange Membrane Fuel Cells with a CsH_5_(PO_4_)_2_-Doped Polybenzimidazole Membrane

**DOI:** 10.3390/ma16113925

**Published:** 2023-05-24

**Authors:** Yizhe Li, Zhiyong Fu, Yifan Li, Guichen Zhang

**Affiliations:** 1 Merchant Marine College, Shanghai Maritime University, Shanghai 201306, China; 202130110106@stu.shmtu.edu.cn; 2 School of Naval Architecture, Ocean & Civil Engineering, Shanghai Jiao Tong University, Shanghai 200240, China; xiaozhenqingnian@sjtu.edu.cn

**Keywords:** catalyst-coated membrane (CCM), membrane electrode assembly (MEA), polymer electrolyte membrane fuel cell (PEMFC)

## Abstract

Membrane electrode assemblies (MEAs) are critical components in influencing the electrochemical performance of high-temperature proton exchange membrane fuel cells (HT-PEMFCs). MEA manufacturing processes are mainly divided into the catalyst-coated membrane (CCM) and the catalyst-coated substrate (CCS) methods. For conventional HT-PEMFCs based on phosphoric acid-doped polybenzimidazole (PBI) membranes, the wetting surface and extreme swelling of the PA-doped PBI membranes make the CCM method difficult to apply to the fabrication of MEAs. In this study, by taking advantage of the dry surface and low swelling of a CsH_5_(PO_4_)_2_-doped PBI membrane, an MEA fabricated by the CCM method was compared with an MEA made by the CCS method. Under each temperature condition, the peak power density of the CCM-MEA was higher than that of the CCS-MEA. Furthermore, under humidified gas conditions, an enhancement in the peak power densities was observed for both MEAs, which was attributed to the increase in the conductivity of the electrolyte membrane. The CCM-MEA exhibited a peak power density of 647 mW cm^−2^ at 200 °C, which was ~16% higher than that of the CCS-MEA. Electrochemical impedance spectroscopy results showed that the CCM-MEA had lower ohmic resistance, which implied that it had better contact between the membrane and catalyst layer.

## 1. Introduction

High-temperature proton exchange membrane fuel cells (HT-PEMFCs) based on phosphoric acid (PA)-doped polybenzimidazole (PBI) membranes have attracted significant attention in the last two decades as promising clean energy sources with the capability to operate at 100–200 °C. Compared with low-temperature PEMFCs, which can operate below 100 °C, HT-PEMFCs have the following advantages due to their higher operating temperature. Water management systems are unnecessary due to the existence of water in the form of vapor in the high-operating-temperature range. HT-PEMFCs exhibit higher tolerance to carbon monoxide because the increased temperature accelerates the desorption process of carbon monoxide on the catalyst surface [1,2]. Furthermore, the rate of the oxygen reduction reaction (ORR) process is enhanced by the increased temperature [3].

Materials and characterization techniques are important for the development of HT-PEMFCs [4,5,6,7,8]. Membrane electrode assemblies (MEAs) are important components that influence the electrochemical performance of HT-PEMFCs. MEAs consist of an electrolyte membrane, two catalyst layers (CLs), and two gas diffusion layers (GDLs). Based on the deposition method of the CL, the manufacturing processes of MEAs are mainly divided into two types. In the first, the GDLs are coated with CLs to form gas diffusion electrodes (GDEs), which are then pressed together with the electrolyte membrane to fabricate the MEA. This is known as the catalyst-coated substrate (CCS) method. In the second, the electrolyte membrane is coated with CLs to form a catalyst-coated membrane (CCM), which is then pressed together with GDLs to obtain the MEA. This is known as the CCM method. The difference between the manufacturing processes of the above two methods can affect the electrochemical properties of MEAs.

The comparison between the CCS and CCM methods used in LT-PEMFCs has been widely reported by many groups [9,10,11,12]. The results showed that the CCM method improved the interfacial contact between the CLs and electrolyte membrane and increased the active sites on the catalyst surface, thereby reducing the interfacial resistance and enhancing the triple phase boundary in the MEAs. However, comparisons between these two MEA fabrication methods used in HT-PEMFCs have rarely been reported due to the fact that some inherent drawbacks of PA-doped PBI membranes make it difficult to apply the CCM method for MEA fabrication. After doping the PBI membrane with PA, the membrane surface is wetted due to the presence of liquid PA, so it is difficult for the CLs to adhere to the membrane surface. Furthermore, even if the CLs are formed by spraying the catalyst ink on the dry surface of the undoped PBI membrane, the subsequent doping with PA causes the PBI membrane to undergo extreme swelling; thus, it is difficult to avoid cracks in the CLs and even peeling of the CLs from the membrane. Although the CCM method is less applicable to MEA fabrication based on the PA-doped PBI membrane, there have still been a few reports. Cho and co-workers [13] reported a CCM method whereby the catalyst ink was sprayed onto a pure PBI membrane to fabricate the MEA, which was then entirely immersed in a PA solution for doping. The performance of the MEA made by this CCM method exhibited a twofold improvement compared with the MEA prepared with the CCS method. Wannek’s group [14,15] reported a method for introducing the PA into an MEA by pre-doping the GDL with PA. In addition, the teams of Shang [16] and Su [17,18] also reported the preparation of MEAs based on the CCM method by introducing PA from a pre-doped GDL. The results showed that the MEAs fabricated by the CCM method exhibited better performances with low Pt loading compared with those made using the CCS method.

Recently, a PBI membrane doped with a molten proton conductor, CsH_5_(PO_4_)_2_, instead of PA, and an MEA based on this membrane fabricated by the CCS method, which can efficiently operate at 220 °C, have been reported [19,20]. Furthermore, the application of this MEA in an internal CuZn-based methanol reformer was also reported [21]. It was found that the surface of the CsH_5_(PO_4_)_2_-doped PBI membrane remained in a dry state at room temperature because CsH_5_(PO_4_)_2_ was solid below its melting point (~150 °C) [21]. In addition, only slight swelling of the PBI membrane occurred after doping with the molten proton conductor [20], implying its suitability for forming the CL by spraying catalyst ink onto the membrane surface. Inspired by these features, in the present study, we fabricated an MEA using the CCM method and evaluated its electrochemical properties. Furthermore, its performance was compared with that of an MEA made by the CCS method. The study showed that, based on the CsH_5_(PO_4_)_2_-doped PBI membrane, the MEA prepared by the CCM method showed a better cell performance compared to the MEA prepared by the CCS method because of the better contact between the membrane and the catalyst layer. This study provides a promising direction for the development of high-performance HT-PEMFCs.

## 2. Experimental Section

### 2.1. CsH_5_(PO_4_)_2_-Doped PBI Membrane Preparation

CsH_5_(PO_4_)_2_ was synthesized using Cs_2_CO_3_ and H_3_PO_4_ according to a method reported previously [19]. The commercial PBI membrane (Fumatech^®^ AP-30) was purchased from FUMATECH BWT GmbH (St. Ingbert, Germany). The CsH_5_(PO_4_)_2_-doped PBI membrane was prepared by immersing the PBI membrane into molten CsH_5_(PO_4_)_2_, as described previously [20]. The thickness of the CsH_5_(PO_4_)_2_-doped PBI membrane was in the range of 38 to 40 μm. The CsH_5_(PO_4_)_2_-doped PBI membrane prepared in this study was the same as that reported in Ref. [20], which has been characterized in detail, including SEM, thermal properties (TGA and DSC), mechanical properties, swelling ratio, and proton conductivity.

### 2.2. Preparation of MEAs Based on CCM and CCS Methods

**Figure 1:**
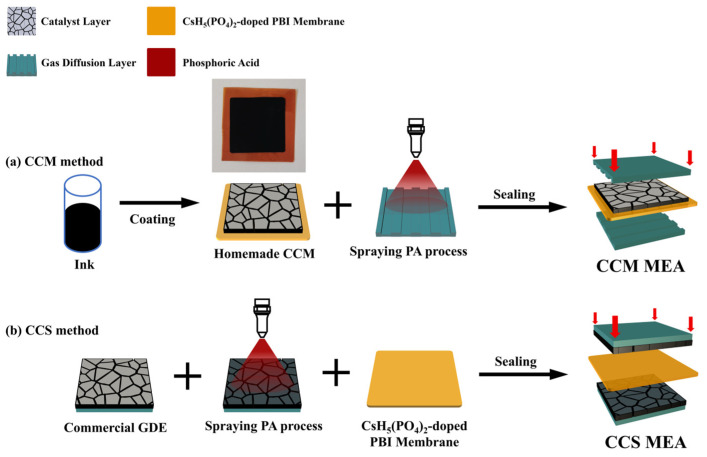
Schematics of preparation process of CCM-MEA and CCS-MEA.

In this study, two types of MEAs based on the CCM and CCS methods were fabricated. A schematic diagram of the MEA preparation process is shown in Figure 1. It is noteworthy that the MEA based on the CsH_5_(PO_4_)_2_-doped PBI membrane needed to have PA introduced into the CLs to compensate for the lack of a proton conductor, as the molten CsH_5_(PO_4_)_2_ cannot easily migrate from the membrane to the CLs due to its high viscosity. The MEA based on the CCM method was prepared by spraying the catalyst ink onto the surface of the CsH_5_(PO_4_)_2_-doped PBI membrane, and then assembled with the GDLs pretreated with PA. The preparation process was as follows. First, to prepare the catalyst ink, 40 wt.% Pt/C catalyst power (Johnson Matthey, London, UK), 5 wt.% polytetrafluoroethylene (PTFE), a PA/ethanol solution, deionized water, and an isopropanol solution in a weight ratio of 1:0.3:2:3:25 were added to a glass beaker and then ultrasonicated under an ice water bath for 1 h. The ethanol/PA solution in catalyst ink was prepared by mixing PA and an ethanol solution in a volume ratio of 1:5, which served to provide proton-conducting channels at the active site in the CLs.

Second, the catalyst ink, prepared as described above, was uniformly coated on the surface of the CsH_5_(PO_4_)_2_-doped PBI membrane on a heating platform at 100 °C by means of a spray gun. The other side of the CsH_5_(PO_4_)_2_-doped PBI membrane was coated with the CL using the same process. The Pt loading of both the anode and cathode CLs was maintained at 1 mg cm^−2^, and the PA remaining in each CL was 1 mg cm^−2^. The active area of the MEA was ~5 cm^2^ (2.25 cm × 2.25 cm). The photo image inserted in Figure 1a shows that the CL-coated membrane was flat, and no visible cracks appeared in the CL. Because the excessive PA contained in the catalyst ink wets the surface of the membrane, the CL cannot easily adhere to the membrane. However, a low PA loading in the CL leads to a deficiency in proton conduction, which can reduce the performance of the MEA. Therefore, additional PA was added to the GDL to compensate for the insufficient PA in the catalyst ink. A commercial GDL (Carbon cloth, Sinero, Suzhou, China, W1S1011) was sprayed with the PA/ethanol solution and then dried in an oven at 120 °C for 40 min to evaporate the ethanol. The residual PA in the GDL was 2 mg cm^−2^, and the other piece of GDL was treated in the same manner. After the GDLs were treated with PA, the CL-coated membrane was sandwiched between two GDLs without hot pressing to make the MEA. For convenience, this kind of MEA was designated as CCM-MEA. Figure 1a shows all the preparation steps for the CCM-MEA.

For comparison, an MEA based on the CCS method was also prepared. The preparation steps are shown in Figure 1b, as reported previously [20]. First, the CL surface of a commercial GDE with Pt loading 1 mg cm^−2^ (Advent, DE, USA, HT 140E) was sprayed with a PA/ethanol solution, and then the GDE was dried in an oven at 120 °C for 40 min. By controlling the time of the spraying PA process, the residual PA in the GDE was limited to 3 mg cm^−2^, which corresponded to the amount of PA remaining in the single-sided electrode of the CCM-MEA. This kind of MEA was designated as CCS-MEA.

### 2.3. Single-Cell Test

The performance and polarization curves of the CCM-MEA and CCS-MEA were evaluated using a commercial fuel cell station (HEPHAS Energy, Guangzhou, China, Mini-M50). The MEA was assembled with two PTFE gaskets and then sandwiched by a carbon bipolar plate to assemble the single-cell hardware. The MEAs were activated at a potentiostatic loading of 0.6 V at 160 °C for at least 10 h by feeding with a gas flow rate of H_2_ and O_2_ at 400 cm min−1 until the output current remained constant. After the activation process, the performance of the single cell was evaluated with the current–voltage (I-V) curve and the current–power (I-P) curve feeding with the dry H_2_/O_2_ to the anode/cathode, respectively, at 160 °C. The feeding gas at the cathode was then switched to 10% relative humidity (RH) gas by pumping the water with the peristaltic pump. Then, the polarization curves were recorded to evaluate the humidifying effects on the performance of the MEAs. After evaluating the performance of the MEAs at 160 °C, the single cell was heated to 180 and 200 °C, respectively, and the same testing process as at 160 °C was repeated.

### 2.4. Electrochemical Characterization of the MEAs

Electrochemical impedance spectroscopy (EIS) and cyclic voltammetry (CV) were evaluated using an electrochemical workstation (Solartron, Leicester, UK, ENERGYLAB XM). During the EIS measurements, the single cell was operated in potentiostatic mode, which was applied at 0.6 V with an amplitude of 10 mV in a frequency range from 10^6^ to 0.1 Hz.

## 3. Results and Discussion

### 3.1. Effects of CCS-MEA and CCM-MEA on Single-Cell Performance

**Figure 2:**
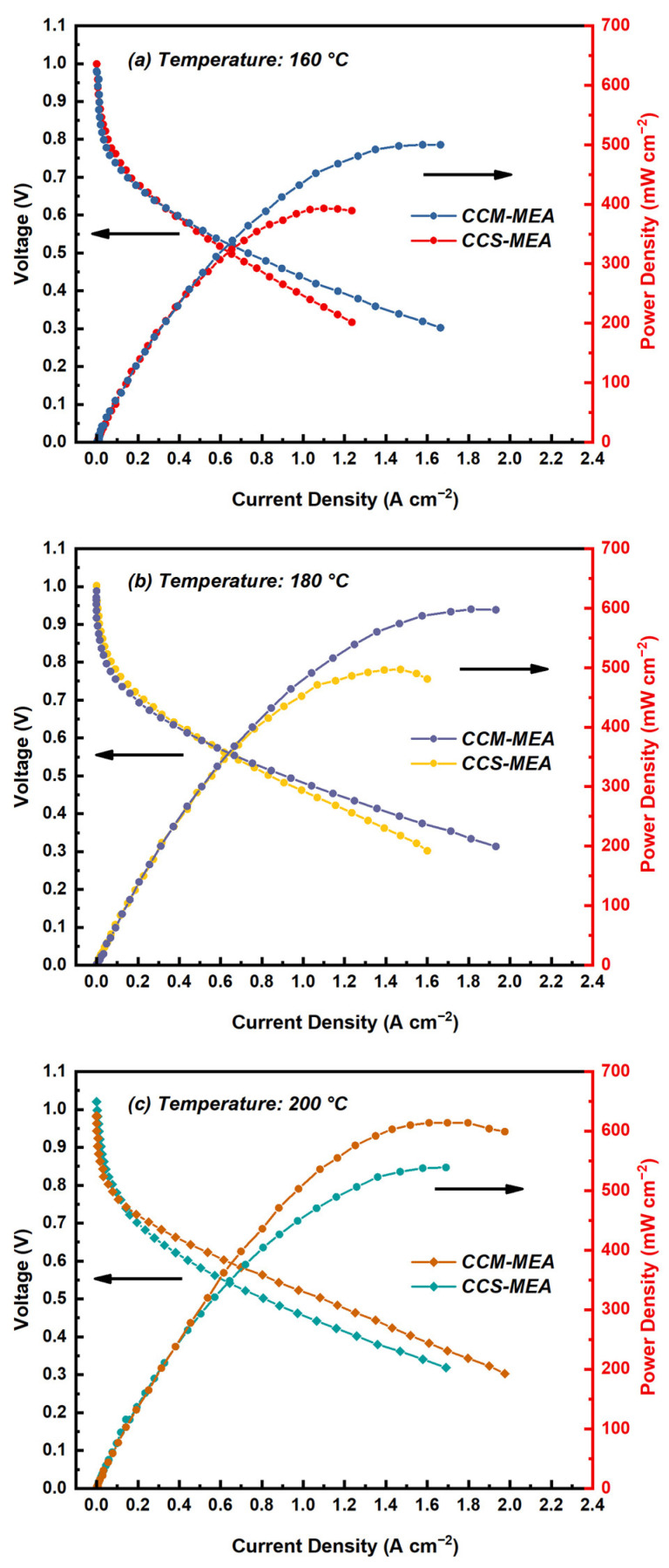
Polarization curves of CCM-MEA and CCS-MEA measured at (**a**) 160 °C, (**b**) 180 °C, and (**c**) 200 °C with dry H_2_ and O_2_.

The performance of single cells of the CCM-MEA and CCS-MEA tested under the temperature range from 160 to 200 °C and fed with both dry hydrogen and oxygen is shown in Figure 2. For the purposes of comparison, the open circuit voltages (OCVs) and peak power densities of the MEAs are presented in Table 1. In the case of the single cell tested at 160, 180, and 200 °C, the corresponding OCVs of the CCM-MEA were 0.989, 0.988 and 0.988 V, respectively, and the corresponding OCVs of the CCS-MEA were 1.00, 1.02, and 1.02 V, respectively. Such high OCVs of the CCM-MEA and CCS-MEA can be attributed to the excellent mechanical properties and the low swelling ratio (25 vol.%) of the CsH_5_(PO_4_)_2_-doped PBI membrane [20]; thus, the hydrogen crossover of the membrane can be reduced. Compared with the CCS-MEA, the CCM-MEA showed slightly lower OCVs, below 1 V, in all temperature conditions. Although the same electrolyte membrane was used for the CCM-MEA and the CCS-MEA, the preparation process of the CCM-MEA, by spraying the catalyst ink onto the membrane surface, could cause the deformation of the membrane surface due to the adsorption of the solvent in the catalyst ink, thus increasing the crossover. Moreover, during the preparation of the catalyst ink, the PA was added to it to provide proton conduction; however, the adsorption of phosphate ions on the Pt/C particles may have reduced the reaction sites on the catalyst’s surface, leading to the lower OCV value [12].

As shown in Figure 2a, the peak power density of the CCM-MEA was 500 mW cm^−2^, which was higher than the 393 mW cm^−2^ achieved by the CCS-MEA under the same measuring conditions. As the cell temperature increased from 160 to 200 °C, the peak power densities of both two types of MEAs increased, which can be ascribed to the combination of the increased reaction kinetics at the CLs [22,23] and the increased conductivities of the CsH_5_(PO_4_)_2_ melted in the membrane due to the increasing temperature [20,24]. At each temperature condition, the peak power density of the CCM-MEA was higher than that of the CCS-MEA. The highest power density value was 614 mW cm^−2^, presented by the CCM-MEA at 200 °C, as shown in Figure 2c. Moreover, as an example for analysis, as shown in Figure 2a, the improved performance of the CCM-MEA compared to the CCS-MEA at 160 °C was ~25%. By comparing the voltage drop behavior of the CCM-MEA and the CCS-MEA in the medium current density region (0.4 to 1.0 A cm^−2^), it can be observed that the voltage drop rate of the CCM-MEA was slower than that of the CCS-MEA, implying that the CCM-MEA had lower ohmic loss. Ohmic loss is usually determined by the ionic resistance of the electrolyte membrane and the contact resistance between the components in the MEA [25]. Because there is no difference in the electrolyte membrane and single-cell hardware used in the two types of MEAs, the lower ohmic loss of the CCM-MEA mainly benefits from the better interfacial contact between the CLs and the electrolyte membrane by directly spraying the catalyst ink onto the membrane surface [11,12]. Moreover, it should be noted that the CCM-MEA presented a faster voltage drop rate compared with the CCS-MEA in the low-current-density region (0 to 0.4 A cm^−2^), as shown in Figure 2a, meaning that the CCM-MEA had a higher charge transfer loss. This is further discussed below. A similarly fast voltage drop rate of the CCM-MEA in the low-current-density region can also be observed in Figure 2b,c.

**Figure 3:**
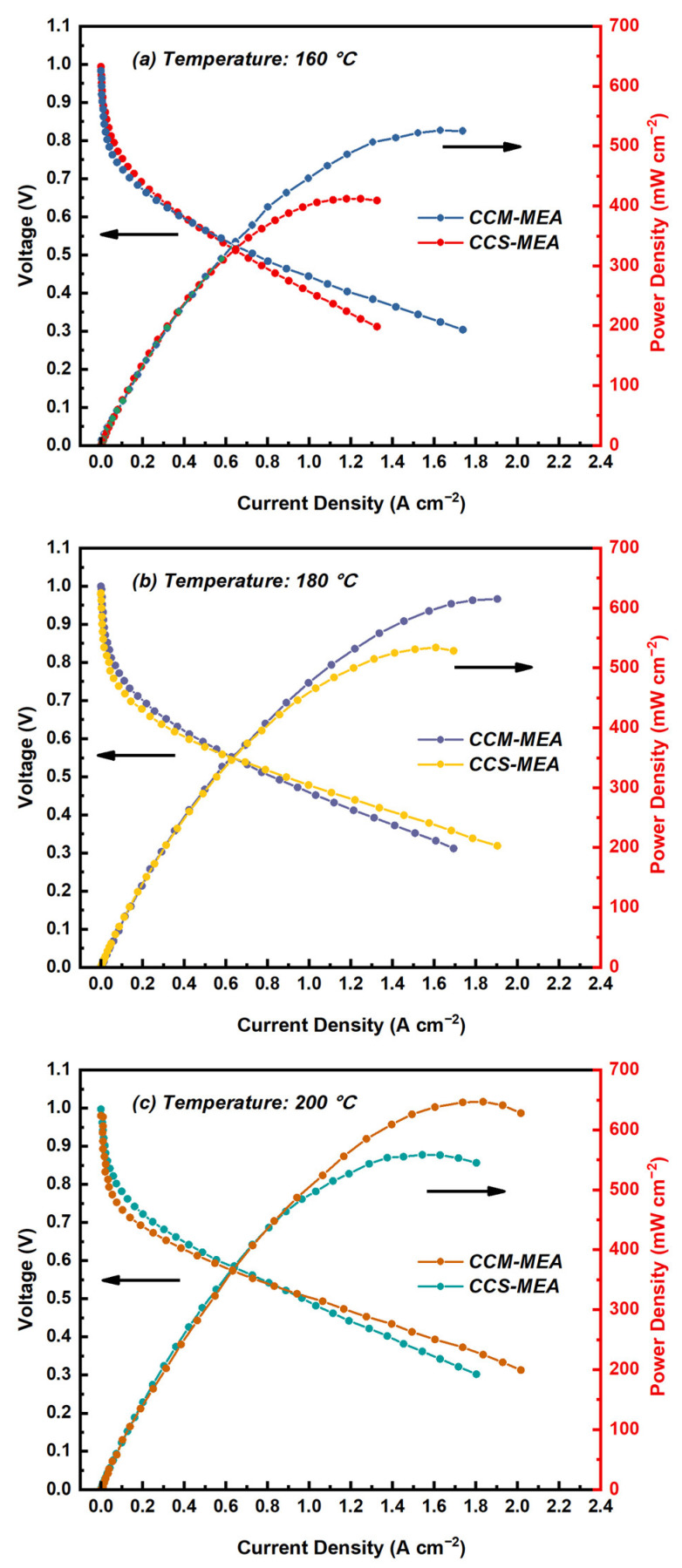
Polarization curves of CCM-MEA and CCS-MEA measured at (**a**) 160 °C, (**b**) 180 °C, and (**c**) 200 °C, with humidifying H_2_ and dry O_2_.

For the HT-PEMFC with the CsH_5_(PO_4_)_2_-doped PBI membrane, humidity is an important factor that affects the MEA performance. A humidified atmosphere not only improves the conductivity of the electrolyte membrane with the molten CsH_5_(PO_4_)_2_ [24,26] and inhibits the decomposition of the CsH_5_(PO_4_)_2_ melt [27], but also maintains a stable output power of MEA during long-term operation [20]. Therefore, it is necessary to evaluate the performance of the molten phosphate electrolyte membrane-based CCM-MEA and CCS-MEA in a humidified atmosphere. Figure 3 shows the performance of the CCM-MEA and CCS-MEA in the humidified atmosphere. In this study, during single-cell operation, only the anode of the single cell was supplied with humidified hydrogen; the cathode was supplied with dry oxygen. This single-electrode humidification method was adopted because it was considered that the cathode of the MEA could be humidified by the water produced through the cell reaction. From Table 1, it can be observed that the OCVs of both the CCM-MEA and CCS-MEA under 10% RH were lower than the equivalent values under dry gas conditions, independent of the operating temperature. According to the Nernst equation, the decrease in the OCVs of the MEAs under humidified gas conditions could be due to the decrease in the hydrogen partial pressure caused by the dilution of feeding hydrogen [22,28].

As shown in Figure 3a, the peak power density of the CCS-MEA at 160 °C was 412 mW cm^−2^. In contrast, the corresponding value (526 mW cm^−2^) of the CCM-MEA was much higher. Compared with the performance of the MEAs under dry gas conditions at 160 °C, as shown in Figure 2a, an enhancement in the peak power densities can be observed for both MEAs under humidified gas conditions, which can be attributed to the increase in the conductivity of the phosphate electrolyte membrane [20]. Similarly, the increase in the performance of the two types of MEAs due to the humidified gas conditions also occurred at 180 and 200 °C, and the performance of the CCM-MEA was better than that of the CCS-MEA at the same temperature conditions. In Table 1, the maximum peak power density of 647 mW cm^−2^ was achieved by the CCM-MEA at 200 °C under humidified gas conditions. Similarly to the voltage drop behavior of both MEAs measured under the dry gas conditions, the charge transfer loss of the CCM-MEA under humidified conditions was slightly higher than that of the CCS-MEA. However, the ohmic loss of the CCM-MEA was lower than that of the CCS-MEA, implying that the difference in voltage drop behavior between both MEAs was independent of the humidified gas conditions.

### 3.2. Effects of CCS-MEA and CCM-MEA on EIS Results

**Figure 4:**
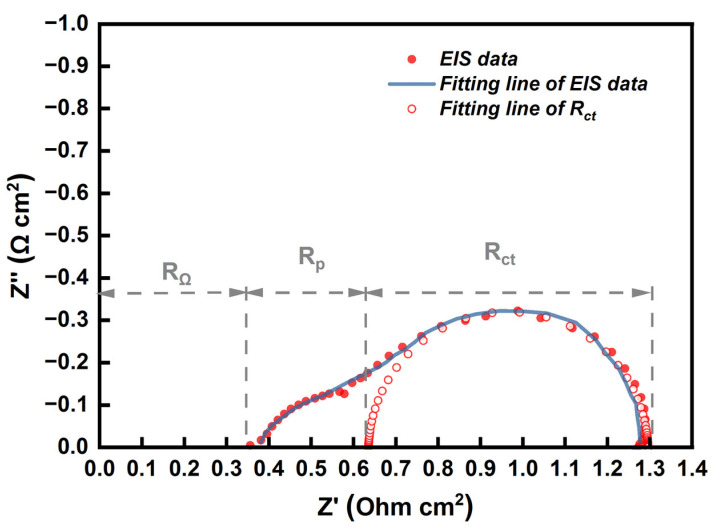
Impedance curve of CCS-MEA measured at 0.6 V and 160 °C (its equivalent circuit and fitting curves are included).

EIS measurements were employed during the single-cell test to better understand the reason for the difference in performance between the CCM-MEA and CCS-MEA. Figure 4 shows the EIS result of the CCS-MEA tested at 160 °C under a potentiostatic mode at 0.6 V, and the equivalent circuit is also presented in Figure 4 to fit the impedance result. In Figure 4, the Nyquist plot is divided into three parts of impedance, namely, the total ohmic resistance (R_Ω_), the proton transfer resistance (R_p_), and the total charge transfer resistance (R_ct_). The total ohmic resistance represents the first intercept value on the Z axis, which is the sum of the ionic resistance of the electrolyte membrane, the inter-contact resistance between the components in the MEA, and the other body resistance of ohmic contributions in the single-cell hardware. Because the ohmic contributions in the single-cell hardware, such as bipolar and current collector plates, remained almost constant, any changes in the total ohmic resistance should be treated as changes in the sum of the ionic resistance of the electrolyte membrane and the inter-contact resistance between the components of the MEA.

In Figure 4, the slope close to 45° appearing in the high-frequency region near the first intercept on the Z′ axis represents the proton transfer loss in the CL. A similar phenomenon has been reported by many groups [29,30,31]; thus, the resistance caused by the proton transfer in CL was deemed the proton transfer resistance. In addition, a semi-circular structure representing the charge transfer loss was observed in the low-frequency region of the Nyquist plot. In this study, the polarization effect of the anode was negligible because the anode and the cathode were supplied with abundant H_2_ and O_2_, respectively. Only a single semi-circle was present in the Nyquist plot, implying that the ORR process dominated the polarization process [32]. The fit of R_ct_ is shown in Figure 4. Due to the presence of proton transfer resistance, the arc in the high-frequency region shows the 45° slope, and the semicircular structure corresponding to R_ct_ is shifted to the right direction along the Z′ axis. The value of R_ct_ could be determined by the diameter of the fit of R_ct_, and the value of R_p_ was determined by the difference between the ohmic resistance and the first intercept of the fit of R_ct_ on the Z′ axis.

**Figure 5:**
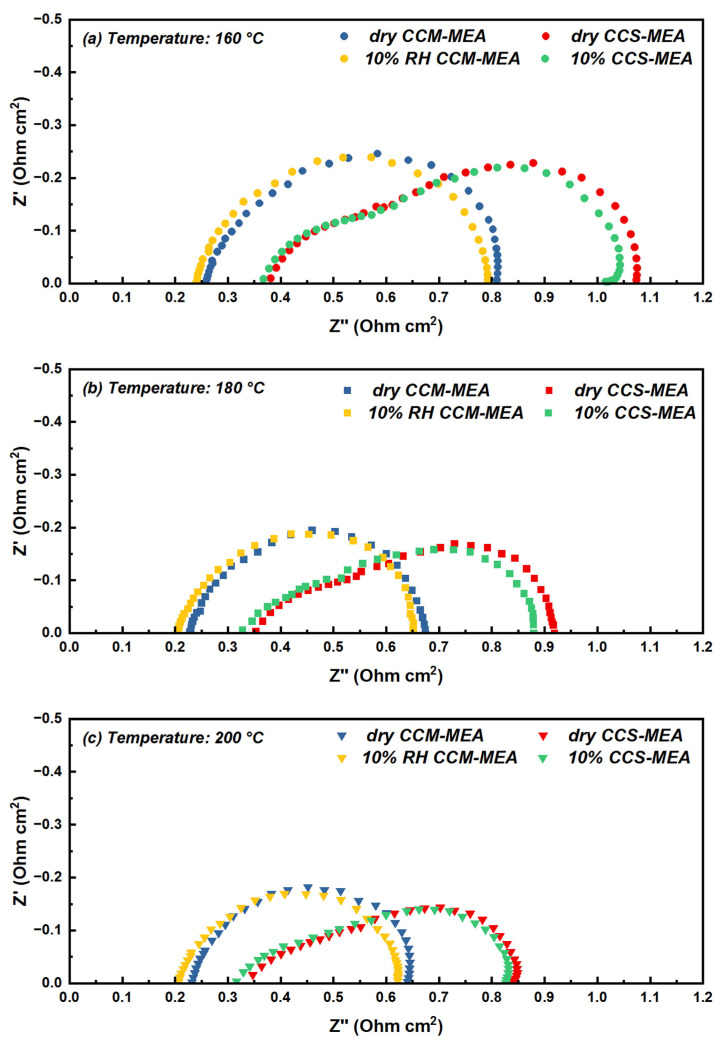
Impedance curves measured for CCS-MEA and CCM-MEA at 0.6 V and (**a**) 160 °C, (**b**) 180 °C, and (**c**) 200 °C under dry gas conditions (feeding with dry H_2_ and O_2_) and humidifying gas conditions (feeding with 10% RH H_2_ and dry O_2_).

**Figure 6:**
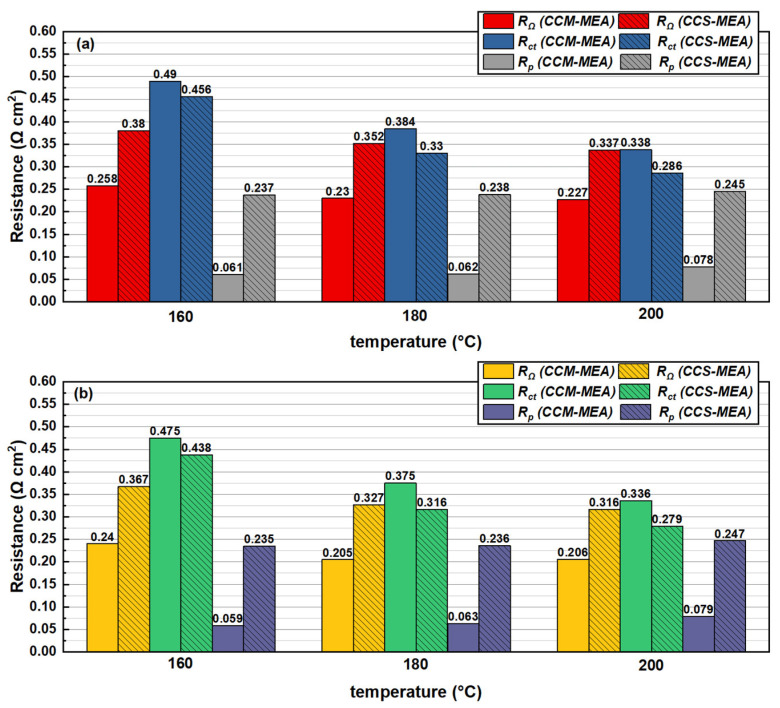
Electrochemical resistance values (R_Ω_, R_ct_ and R_p_) of CCM-MEA and CCS-MEA measured at 0.6 V from 160 to 200 °C, feeding with (**a**) dry H_2_ and dry O_2_ and (**b**) 10% RH H_2_ and dry O_2_, respectively.

The EIS results of the CCM-MEA and CCS-MEA measured at 0.6 V under different temperature conditions are shown in Figure 5, and the effects of humidifying fuel on the EIS results are also presented. To clearly illustrate thus, the values of R_Ω_, R_ct_, and R_p_ obtained from Figure 5 are shown in Figure 6. It can be observed that the Nyquist plots of the CCM-MEA all presented with a single semi-circle structure. This indicates that the effect of proton transfer resistance at the CL was weak for the CCM-MEA, and the main polarization effect was dominated by the ORR process independently of the current density. In contrast, the EIS results of the CCS-MEA showed that the effect of proton transfer loss was obvious in the high-frequency region of the Nyquist plots, implying that the R_p_ of the CCS-MEA was greater than that of the CCM-MEA.

In the case of the impedance curve tested under dry gas conditions at 160 °C, as shown in Figure 5a, it can be observed that the R_Ω_ value of the CCM-MEA (0.258 Ω cm^2^) was lower than that of the CCS-MEA (0.38 Ω cm^2^), implying that coating the catalyst ink on the surface of the electrolyte membrane to form the CL led to better interfacial contact between the CLs and the membrane. In Figure 5a, by comparing the shape of the impedance curve in the high-frequency region between the CCM-MEA and CCS-MEA, it can be seen that the shape of the CCS-MEA is closer to the 45° slope, resulting in a more significant effect of proton transfer limitation of the CL in the CCS-MEA. This phenomenon could be ascribed to the different PA introduction methods for the CCS-MEA and CCM-MEA, as shown in Figure 1. For the preparation of the CCM-MEA, part of the PA/ethanol mixture was ultrasonically dispersed in the catalyst ink before it was sprayed onto the membrane surface. The spraying process was conducted on the heating platform to evaporate the alcoholic solvent during the formation of the CL so that this process could ensure that the PA was evenly distributed and entirely retained in the CL. However, for the preparation of the CCS-MEA, the PA/ethanol mixture was sprayed onto the CL surface of the commercial GDE, and the PA/ethanol mixture tended to diffuse from the CL surface to the CL/GDL interface, thus leading to an inhomogeneous PA distribution in the CL. The inhomogeneous proton conductor distributed in the CL affected the shape of the impedance curve in the high-frequency region, as was studied by Malevich et al. [33]. In contrast, in the medium- and low-frequency regions, the shapes of the impedance curve of the CCM-MEA and CCS-MEA both presented semi-circle structures, and the R_ct_ value of the CCM-MEA was slightly higher than that of the CCS-MEA, meaning that the CCM-MEA has lower reaction kinetics for the ORR process. In Figure 3, the voltage drop behavior of the CCM-MEA, showing a faster voltage drop in the high-voltage region compared to the CCS-MEA, also reflects that the ORR process of the CCM-MEA was slower than that of the CCS-MEA. As discussed above, during the preparation of the catalyst ink, the PA was added to the catalyst ink to provide the proton conductor, but the adsorption of phosphate ions on the Pt/C particles may have reduced the reaction sites on the catalyst surface, leading to higher charge transfer resistance.

To further understand the reason why the CCM-MEA presented better performance at 180 and 200 °C, the EIS results of the CCM-MEA operating at 180 and 200 °C are shown in Figure 5b,c, respectively, and the EIS results of the CCS-MEA are also shown for comparison. When the CCM-MEA operated at 180 °C, the R_Ω_ value of the CCM-MEA was 0.230 Ω cm^2^, lower than the R_Ω_ value of the CCM-MEA collected at 160 °C, as shown in Figure 6. The decrease in R_Ω_ by increasing the operation temperature from 160 to 180 °C could be ascribed to the increase in the conductivity of the molten proton conductor in the membrane with the increasing temperature [19]. Similarly, the R_Ω_ value of the CCM-MEA further decreased to 0.227 Ω cm^2^ as the temperature increased to 200 °C. Moreover, the EIS results of the CCM-MEA measured under humidifying gas conditions clearly show that the R_Ω_ decreased as the feeding gas was switched from dry hydrogen to 10% RH hydrogen. This decrease in R_Ω_ under humidity gas conditions could be due to the hygroscopicity of CsH_5_(PO_4_)_2_, which increased the proton conductivity of the CsH_5_(PO_4_)_2_-doped PBI membrane. This could be the reason that both the CCM-MEA and CCS-MEA showed better performances in humidifying gas conditions, as shown in Figure 3.

Furthermore, comparing the impedance curves of the CCM-MEA at 160 and 180 °C, it can be clearly seen that the CCM-MEA at 180 °C presented a smaller semi-circle structure and a lower R_ct_ value. This can be attributed to the fact that, according to the Bulter–Volmer equation, an increase in temperature induces an increase in the reaction kinetics of the ORR process [22]; therefore, the R_ct_ value of CCM-MEA decreases with the increasing temperature. The change in R_p_ with the increasing temperature is different to that with R_Ω_ and R_ct_. The R_p_ (0.061 Ω cm^2^) of the CCM-MEA measured at 160 °C was similar to the R_p_ (0.062 Ω cm^2^) measured at 180 °C, but when the temperature increased from 180 to 200 °C, the R_p_ value increased to 0.078 Ω cm^2^. This sudden increase in R_p_ due to the temperature increase to 200 °C was also observed in the CCS-MEA, as the R_p_ shows in Figure 6. This can be attributed to the PA in the CLs possibly undergoing dehydration at 200 °C under the dry gas conditions, leading to the formation of pyrophosphate with low conductivity. The R_p_ values of both the CCM-MEA and CCS-MEA measured at 10% RH humidity conditions were not significantly different from those measured under dry conditions, implying that the limited humidified environment could not inhibit PA dehydration at 200 °C.

## 4. Conclusions

In this study, MEAs based on the CsH_5_(PO_4_)_2_-doped PBI membrane were prepared by the CCM and CCS methods, and their cell performance under 160–200 °C feeding with H_2_/O_2_ was evaluated by polarization curves and EIS. Both types of MEAs were able to maintain OCVs of ~1 V. Under feeding with both dry hydrogen and oxygen, at each temperature condition, the peak power density of the CCM-MEA was higher than that of the CCS-MEA. Furthermore, under humidified gas conditions, an enhancement in the peak power densities could be observed for both MEAs, which may be attributed to the increase in the conductivity of the electrolyte membrane. The performance of the CCM-MEA was higher than that of the CCS-MEA at the same temperature conditions. EIS was used to investigate the possible reason for the better performance of the CCM-MEA. The results showed that the CCM-MEA had lower ohmic resistance, implying that the MEA prepared by the CCM method had better contact between the membrane and catalyst layer. In addition, the better performance of the CCM-MEA was also attributed to the lower proton transfer resistance in the catalyst layer.

## Figures and Tables

**Table 1:** Summary of electrochemical performance (OCV and peak power densities) of CCM-MEA and CCS-MEA with different gas conditions.

MEA Type	Temperature (°C)	Gas Conditions at the Anode	Open Circuit Voltage (V)	Peak Power Density (mW cm^−2^)
CCS-MEA	160	dry	1.000	393
		10% RH	0.994	412
	180	dry	1.02	497
		10% RH	0.992	534
	200	dry	1.020	537
		10% RH	0.997	558
CCM-MEA	160	dry	0.989	500
		10% RH	0.983	526
	180	dry	0.988	598
		10% RH	0.982	613
	200	dry	0.988	614
		10% RH	0.980	617

## Data Availability

Not applicable.
